# Severe Thrombocytopenia in Actinotignum schaalii Bacteremia: A Case Report With Review of the Literature

**DOI:** 10.7759/cureus.114695

**Published:** 2026-08-17

**Authors:** Alireza Izadian Bidgoli, Alberto Gomez Veliz, Angela C Gallagher, Hellen Ailette Gomez Usatorres, Alberto Gomez Usatorres

**Affiliations:** 1 Internal Medicine, American University of the Caribbean School of Medicine, Cupecoy, SXM; 2 Internal Medicine, Jackson Memorial Hospital, Miami, USA; 3 Internal Medicine, Universidad Hispanoamerica, San Jose, CRI

**Keywords:** actinotignum schaalii, disseminated intravascular coagulation, sepsis, thrombocytopenia, thrombotic microangiopathy

## Abstract

*Actinotignum schaalii* is an emerging Gram-positive uropathogen that primarily affects older adults with underlying genitourinary abnormalities and can rarely present with severe systemic manifestations. We report the case of a 73-year-old man with a chronic suprapubic catheter who presented with fever, generalized weakness, severe thrombocytopenia, acute kidney injury, and systemic inflammation. Blood cultures grew *A. schaalii*, while the combination of profound thrombocytopenia and renal dysfunction initially raised concern for disseminated intravascular coagulation (DIC), thrombotic thrombocytopenic purpura, hemolytic uremic syndrome, and other thrombotic microangiopathies. Comprehensive hematologic evaluation, including peripheral blood smear, coagulation studies, hemolysis markers, ADAMTS13 activity, and platelet recovery with treatment of the underlying infection, excluded these competing diagnoses and supported sepsis-associated thrombocytopenia. Computed tomography, renal ultrasonography, and technetium-99m MAG3 diuretic renography demonstrated severe chronic left hydronephrosis with marked cortical thinning and only 14.5% differential renal function, consistent with chronic irreversible obstructive nephropathy rather than acute bilateral renal injury. The patient improved with targeted antimicrobial therapy and supportive management, with resolution of bacteremia, recovery of renal function, and normalization of platelet count. This case highlights the importance of maintaining a broad differential diagnosis for severe thrombocytopenia during bacteremia and integrating microbiologic, hematologic, and radiographic findings to distinguish sepsis-associated thrombocytopenia from life-threatening thrombotic and consumptive disorders, thereby avoiding unnecessary interventions while ensuring timely, appropriate management.

## Introduction

Actinotignum schaalii (formerly Actinobaculum schaalii) is an emerging Gram-positive facultative anaerobic bacillus increasingly recognized as a cause of urinary tract infections and bacteremia, particularly among older adults with underlying genitourinary abnormalities, chronic urinary catheterization, or obstructive uropathy [1,2]. Because of its slow growth and fastidious culture requirements, the organism is frequently underdiagnosed using conventional microbiologic techniques, resulting in delayed recognition and an underestimation of its clinical significance [1,2]. Although A. schaalii is an established urinary pathogen, reports describing severe systemic infection complicated by profound thrombocytopenia remain limited [1,3].

Thrombocytopenia is a common hematologic abnormality in patients with sepsis and is associated with increased morbidity and mortality [4,5]. However, distinguishing sepsis-associated thrombocytopenia from disseminated intravascular coagulation (DIC), thrombotic microangiopathies (TMAs) such as thrombotic thrombocytopenic purpura (TTP) and hemolytic uremic syndrome (HUS), or immune thrombocytopenia (ITP) remains a major clinical challenge because these disorders often present with overlapping clinical and laboratory features while requiring markedly different therapeutic approaches [4-6]. Careful integration of microbiologic, hematologic, coagulation, and radiographic findings is therefore essential to establish the correct diagnosis and avoid unnecessary or potentially harmful interventions [4,6]. We present a case of A. schaalii bacteremia complicated by severe thrombocytopenia and acute kidney injury in which differentiating among DIC, TMA, ITP, and sepsis-associated thrombocytopenia required a comprehensive multidisciplinary evaluation.

## Case presentation

Clinical presentation

A 73-year-old Hispanic man with a medical history of hypertension, type 2 diabetes mellitus, hyperlipidemia, benign prostatic hyperplasia complicated by chronic urinary retention requiring a long-term suprapubic catheter, chronic left hydronephrosis, and previously documented thrombocytopenia presented to the emergency department with progressive generalized weakness, fatigue, and decreased urine output over several days. The patient denied fever, chills, nausea, vomiting, abdominal pain, diarrhea, melena, hematochezia, hematemesis, hematuria, epistaxis, gingival bleeding, or recent trauma. He also denied chest pain, dyspnea, cough, headache, visual disturbances, or focal neurological deficits. His family reported increasing lethargy and reduced oral intake prior to presentation.

His medical history was notable for chronic thrombocytopenia that had been monitored in the outpatient setting without a definitive diagnosis. He had no known history of autoimmune disease, malignancy, liver disease, or prior TMA. His medications included antihypertensive therapy, statin therapy, medications for benign prostatic hyperplasia, and oral hypoglycemic agents. There was no recent exposure to heparin, chemotherapy, or other medications commonly associated with drug-induced thrombocytopenia.

Because of the combination of profound thrombocytopenia, acute kidney injury, systemic inflammation, and concern for sepsis, he was admitted for further evaluation and management.

Initial assessment and physical examination

On arrival, the patient appeared chronically ill and fatigued but remained alert and oriented without acute distress. Initial vital signs demonstrated hemodynamic stability without persistent hypotension. Cardiopulmonary examination was unremarkable with normal heart sounds and clear breath sounds bilaterally. Abdominal examination was soft and non-distended without tenderness or guarding. The suprapubic catheter was in place without surrounding erythema or purulent drainage.

No petechiae, purpura, ecchymoses, or mucosal bleeding were identified despite severe thrombocytopenia. There was no scleral icterus or clinical evidence of overt hemolysis. Neurological examination revealed no focal motor or sensory deficits, and mental status remained intact throughout the initial evaluation.

The absence of clinically significant bleeding despite a critically reduced platelet count contrasted with the severity of the laboratory abnormalities and contributed to the diagnostic uncertainty.

Laboratory and diagnostic testing

Initial laboratory evaluation revealed marked leukocytosis (15.9 ×10³/µL) with neutrophilic predominance, severe thrombocytopenia with a platelet count of 16 ×10³/µL, and mild normocytic anemia. Renal function testing demonstrated acute kidney injury with a serum creatinine of 2.40 mg/dL, blood urea nitrogen of 73 mg/dL, and an estimated glomerular filtration rate of approximately 28 mL/min/1.73 m². Serum lactate was elevated at 3.6 mmol/L, while inflammatory markers were markedly increased, including a C-reactive protein of 23.1 mg/dL and erythrocyte sedimentation rate of 86 mm/hr (Table 1).

**Table 1:** Laboratory and Microbiological Findings Laboratory values at hospital admission, peak or most clinically significant abnormalities during hospitalization, and at discharge are shown. The patient's course was characterized by severe thrombocytopenia with a markedly elevated immature platelet fraction, acute kidney injury, hyperbilirubinemia, elevated inflammatory markers, and coagulation abnormalities that initially raised concern for disseminated intravascular coagulation (DIC), thrombotic microangiopathy, and other thrombotic thrombocytopenic syndromes. Blood cultures obtained on admission grew Actinotignum schaalii (formerly Actinobaculum schaalii) in two of two culture sets, while urine cultures remained negative. Abbreviations: ALT, alanine aminotransferase; AST, aspartate aminotransferase; aPTT, activated partial thromboplastin time; BUN, blood urea nitrogen; CRP, C-reactive protein; eGFR, estimated glomerular filtration rate; ESR, erythrocyte sedimentation rate; FEU, fibrinogen equivalent units; HIV, human immunodeficiency virus; INR, international normalized ratio; IPF, immature platelet fraction; LDH, lactate dehydrogenase; PT, prothrombin time.

Laboratory Test	Admission	Peak/Worst During Hospitalization	Discharge	Reference Range / Unit
White blood cell count	15.9	19.1	12.8	4.0-11.0 ×10³/µL
Hemoglobin	12.6	8.8	8.8	13.5-17.5 g/dL
Hematocrit	35.4	26.4	26.4	41-53 %
Platelet count	16	341	341	150-450 ×10³/µL
Immature platelet fraction (IPF)	39	39	19	1-7 %
Absolute neutrophil count	15.3	15.3	9.4	1.8-7.7 ×10³/µL
Absolute lymphocyte count	0.2	0.2	1.5	1.0-4.8 ×10³/µL
ESR	86	86	-	0-20 mm/hr
C-reactive protein	23.1	23.1	-	<0.5 mg/dL
Lactate	3.6	3.6	1.7	0.5-2.0 mmol/L
Creatinine	2.40	2.40	0.81	0.70-1.30 mg/dL
Blood urea nitrogen	73	73	15	7-20 mg/dL
eGFR	28	28	>90	≥60 mL/min/1.73 m²
Total bilirubin	3.3	3.3	1.3	0.2-1.2 mg/dL
AST	70	83	60	10-40 U/L
ALT	38	72	51	7-56 U/L
LDH	257	257	-	140-280 U/L
Haptoglobin	289	289	-	30-200 mg/dL
PT	16.6	16.6	-	11-14 sec
INR	1.32	1.32	-	0.8-1.2
aPTT	38	38	-	25-35 sec
Fibrinogen	551	551	-	200-400 mg/dL
D-dimer	12.53	12.53	-	<0.50 µg/mL FEU
Blood cultures	Gram-positive rods in 2/2 bottles	Actinotignum schaalii isolated in 2/2 sets	Completed treatment	Negative
Urine culture	No growth	No growth	No growth	No growth
Hepatitis B surface antigen	Negative	-	Negative	Negative
Hepatitis C antibody	Negative	-	Negative	Negative
HIV Ag/Ab	Negative	-	Negative	Negative

Peripheral blood smear demonstrated no abnormalities. The immature platelet fraction was markedly elevated at 39%, suggesting preserved bone marrow platelet production. Lactate dehydrogenase was only mildly elevated, haptoglobin remained within the normal range, bilirubin was not significantly increased, and the reticulocyte response was not consistent with ongoing severe microangiopathic hemolysis. Coagulation studies demonstrated only mild abnormalities without the degree of consumptive coagulopathy typically expected in overt DIC, and fibrinogen levels remained preserved. Collectively, these findings failed to clearly support either DIC or TMA despite the profound thrombocytopenia and acute kidney injury.

Comprehensive infectious evaluation was initiated. Urinalysis demonstrated marked pyuria, hematuria, positive leukocyte esterase, proteinuria, and abundant bacteria, consistent with a complicated urinary tract infection. Urine culture demonstrated no bacterial growth after incubation. Blood cultures from two separate sets subsequently grew gram-positive rods in both anaerobic bottles, which were ultimately identified as Actinotignum schaalii (formerly Actinobaculum schaalii), an uncommon uropathogen associated with chronic urinary tract abnormalities and catheter-associated infections. Screening for hepatitis B, hepatitis C, and human immunodeficiency virus was negative (Table 1).

**Figure 1:**
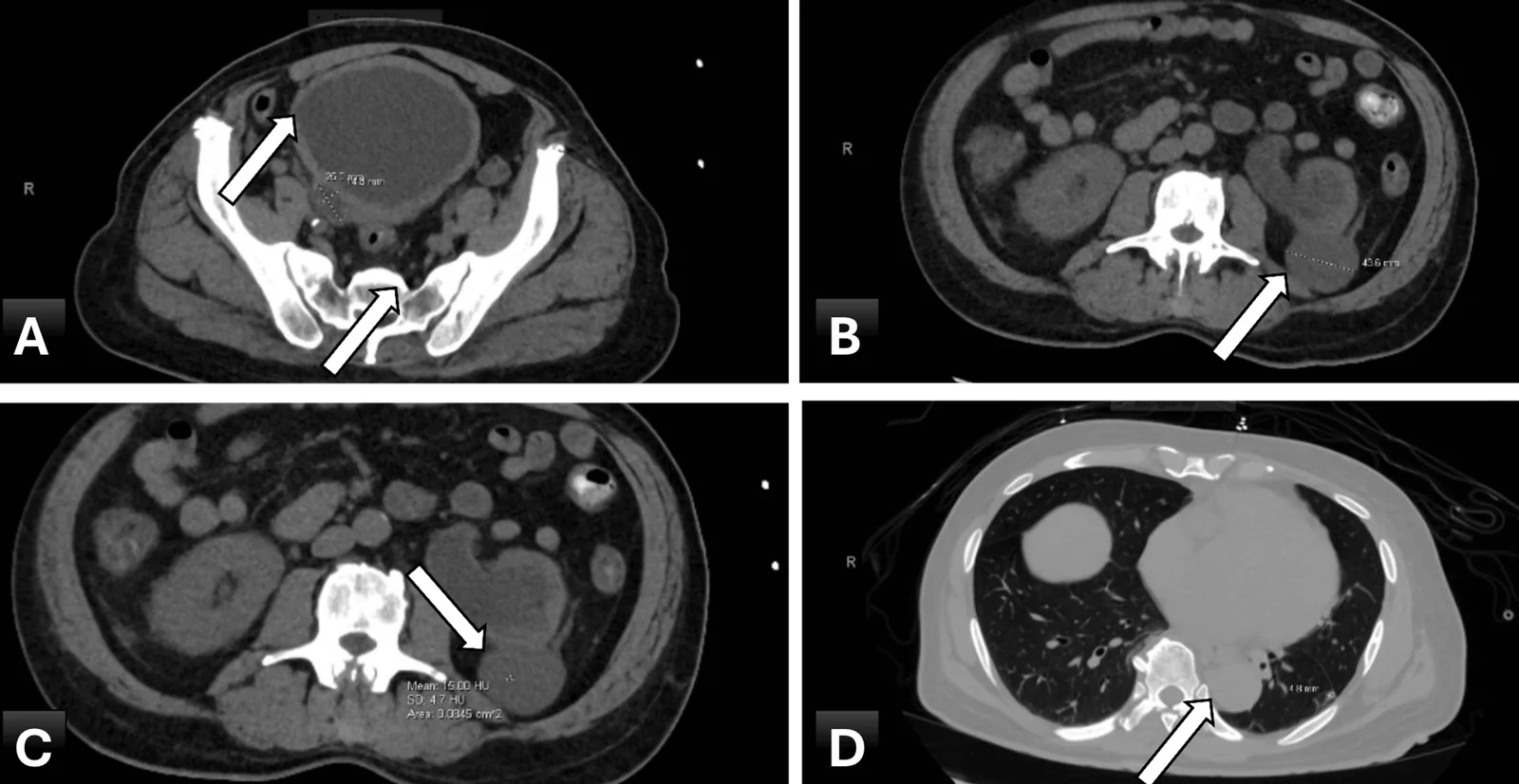
Computed tomography of the abdomen and pelvis demonstrating chronic obstructive uropathy and associated urinary tract abnormalities. (A) Axial CT image demonstrating marked bladder distention with diffuse circumferential bladder wall thickening and trabeculation (upper arrow) in the setting of chronic bladder outlet obstruction. The suprapubic catheter is also visible (lower arrow). (B) Axial CT image demonstrating a simple left lower pole renal cyst (arrow). (C) Axial CT image showing severe chronic left hydroureteronephrosis with marked renal cortical thinning (arrow), consistent with longstanding obstructive nephropathy. (D) Lung window image demonstrating a small left lower lobe perifissural pulmonary nodule (approximately 5 mm, arrow), considered an incidental finding.

Computed tomography (CT) of the abdomen and pelvis demonstrated severe chronic left hydroureteronephrosis with marked renal cortical thinning, associated left perinephric and periureteral inflammatory fat stranding concerning for superimposed ascending urinary tract infection/pyelonephritis. Additional findings included a 4.4-cm simple left lower pole renal cyst, circumferential bladder wall thickening with trabeculation surrounding the suprapubic catheter, and prostatomegaly, suggesting chronic bladder outlet obstruction, although the unilateral nature of the hydronephrosis raised concern for an additional chronic distal left ureteral or ureterovesical junction obstruction. No obstructing urinary tract calculi were identified (Figure 1).

**Figure 2:**
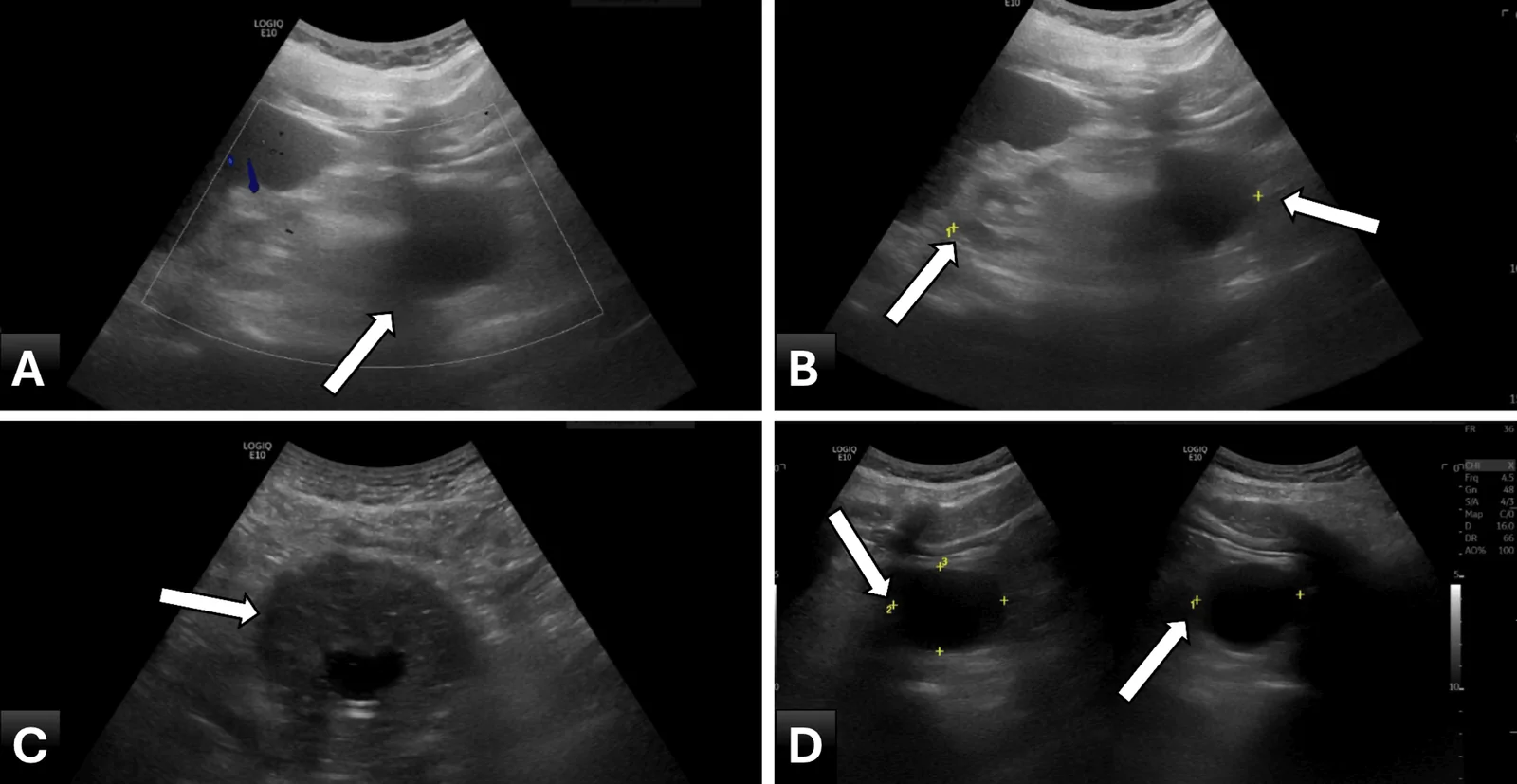
Renal ultrasonography demonstrating chronic obstructive uropathy of the left kidney (A) Longitudinal view demonstrating a simple left lower pole renal cyst (arrow). (B) Left kidney demonstrating severe grade IV hydronephrosis with marked cortical thinning (arrows). (C) Transverse bladder view showing diffuse concentric bladder wall thickening (arrow), consistent with chronic bladder outlet obstruction and/or chronic cystitis. (D) Additional orthogonal view of the simple left lower pole renal cyst (arrow).

Renal ultrasonography further characterized these abnormalities by confirming grade IV left hydronephrosis with marked cortical thinning, a simple lower pole renal cyst, and diffuse concentric bladder wall thickening, while the right kidney appeared normal (Figure 2).

**Figure 3:**
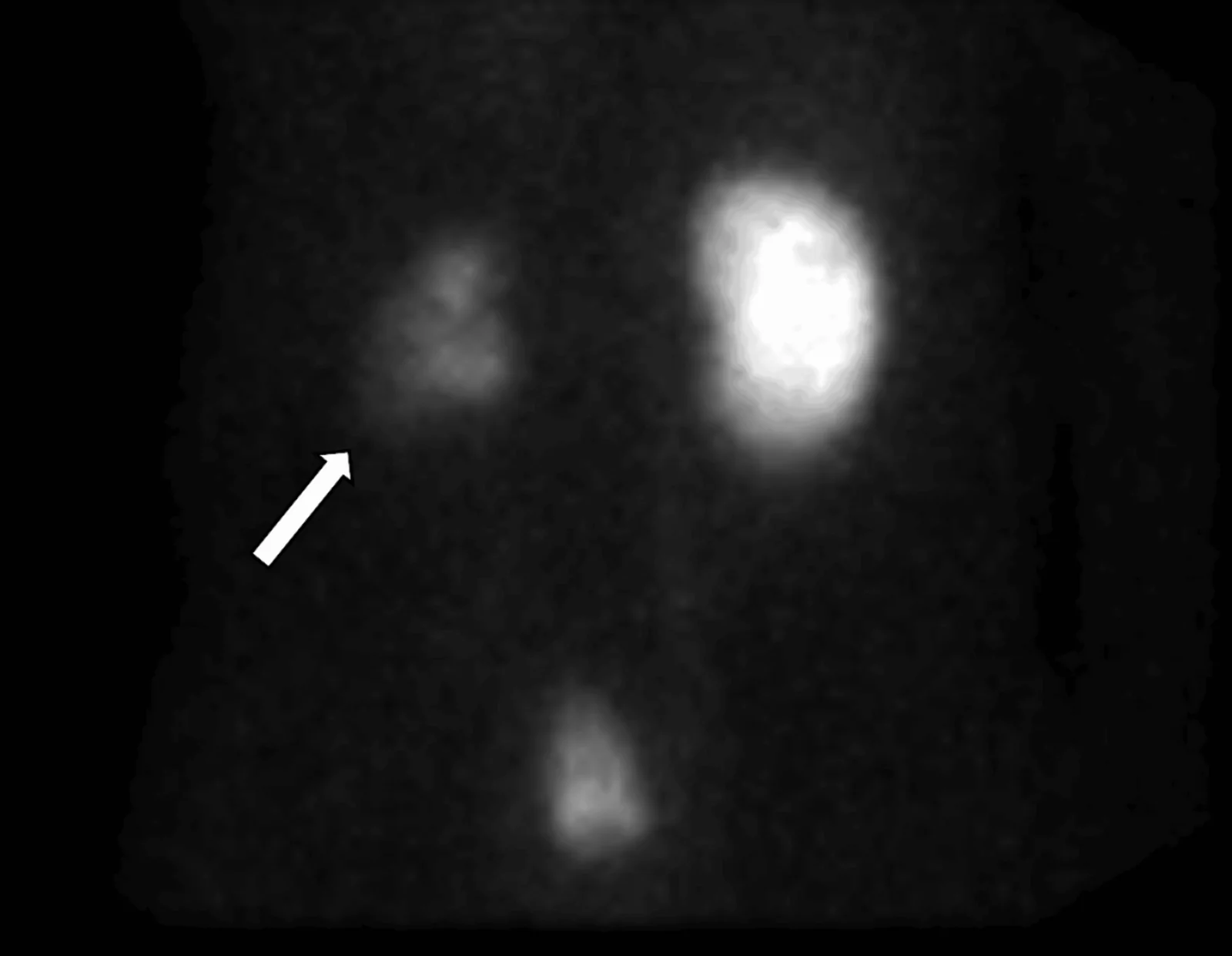
Technetium-99m MAG3 diuretic renogram demonstrating markedly asymmetric renal function. Posterior projection obtained during MAG3 renal scintigraphy shows markedly reduced radiotracer uptake in the left kidney (arrow), consistent with severely impaired perfusion and cortical function. Quantitative analysis demonstrated 14.5% differential function of the left kidney compared with 85.5% for the right kidney, supporting chronic irreversible obstructive nephropathy.

Although CT and renal ultrasonography established the structural abnormalities of the left kidney, the degree of residual renal function and the contribution of chronic obstructive nephropathy to the patient's renal dysfunction remained uncertain. This distinction was clinically relevant because the concurrent acute kidney injury and profound thrombocytopenia had raised concern for TMA. Therefore, a technetium-99m MAG3 diuretic renogram was obtained to quantify differential renal function and further characterize the functional significance of the chronic left-sided obstruction. The study demonstrated markedly diminished perfusion and cortical tracer uptake of the left kidney, which contributed only 14.5% of total differential renal function, compared with 85.5% from the right kidney, which showed preserved cortical uptake but delayed radiotracer clearance (Figure 3). These findings confirmed severe chronic left renal dysfunction and helped establish that a substantial component of the renal abnormality reflected longstanding irreversible obstructive nephropathy rather than acute bilateral renal injury.

Given the constellation of severe thrombocytopenia, acute kidney injury, systemic inflammation, and Actinotignum schaalii bacteremia, the differential diagnosis remained broad and included DIC, TTP, HUS, ITP, and sepsis-associated thrombocytopenia.

Multidisciplinary board discussion

The patient's profound thrombocytopenia in combination with acute kidney injury prompted consultation with Hematology early during hospitalization. Initial concern centered on TMA, particularly TTP and HUS, because of the coexistence of renal dysfunction and severe thrombocytopenia. However, the absence of significant schistocytosis on peripheral smear, normal haptoglobin concentration, only minimal lactate dehydrogenase elevation, preserved bilirubin levels, and lack of laboratory evidence supporting ongoing microangiopathic hemolysis argued against these diagnoses.

DIC was also considered because the patient presented with bacteremia and systemic inflammation. Nevertheless, coagulation abnormalities were relatively modest, fibrinogen remained preserved, and there was no clinical evidence of diffuse bleeding or consumptive coagulopathy, making overt DIC less likely.

The markedly elevated immature platelet fraction suggested appropriate marrow compensation with increased peripheral platelet destruction rather than impaired platelet production. Given the patient's history of chronic thrombocytopenia predating the current infectious episode and the absence of convincing laboratory evidence supporting either DIC or TMA, the hematology service considered immune-mediated thrombocytopenia to be the leading diagnosis, although infection-related thrombocytopenia remained an important contributing factor. Because the patient's platelet count was critically low and multiple potentially life-threatening etiologies had to be excluded simultaneously, treatment decisions were made collaboratively between the hematology, infectious diseases, nephrology, and hospital medicine teams.

Diagnosis and management

The patient was initially managed for a complicated catheter-associated urinary tract infection with gram-positive bacteremia and sepsis. Empiric broad-spectrum antimicrobial therapy with cefepime 1 g intravenously every 12 hours was initiated. Following blood cultures demonstrating gram-positive rods, intravenous vancomycin was added with pharmacy-guided dosing; a regimen of 750 mg intravenously every 12 hours was documented, with a trough concentration of 11.7 µg/mL during therapy. Final microbiologic identification subsequently demonstrated Actinotignum schaalii bacteremia.

Aggressive intravenous fluid resuscitation was initiated for treatment of sepsis-associated acute kidney injury, resulting in progressive improvement in renal function throughout hospitalization. The suprapubic catheter was maintained and closely monitored, while serial laboratory studies were obtained to evaluate for evolving DIC or TMA.

Given persistent severe thrombocytopenia and the lack of laboratory evidence supporting TTP, HUS, or overt DIC, the patient was treated with high-dose corticosteroid therapy for presumed ITP. Platelet transfusion was reserved for severe thrombocytopenia because of the uncertainty surrounding the underlying etiology and concern for limited efficacy if immune-mediated destruction were present. Serial hematologic studies were performed to monitor platelet recovery, renal function, coagulation parameters, and evidence of hemolysis throughout hospitalization.

Clinical outcome and follow-up 

The patient's clinical condition improved steadily following initiation of antimicrobial therapy, supportive care, and corticosteroid treatment. Leukocytosis and inflammatory markers progressively normalized, while serum creatinine decreased from 2.40 mg/dL on admission to 0.81 mg/dL by discharge, consistent with resolution of sepsis-associated acute kidney injury. Blood cultures cleared following treatment, and no additional episodes of bacteremia were documented.

Most notably, the platelet count demonstrated a sustained and progressive recovery from 16 ×10³/µL at presentation to 341 ×10³/µL before discharge. This robust response to corticosteroid therapy strongly supported immune-mediated peripheral platelet destruction as the principal mechanism of thrombocytopenia rather than DIC or TMA. Throughout hospitalization, the patient experienced no clinically significant bleeding, thrombotic complications, or neurological deterioration.

He was discharged home in stable condition with improving renal function, normalization of platelet count, and plans for close outpatient follow-up with hematology, infectious diseases, nephrology, and urology. Ongoing management included completion of antimicrobial therapy, continued monitoring of platelet counts, and long-term evaluation of his chronic obstructive uropathy and severely reduced left renal function.

## Discussion

Background****

Thrombocytopenia is a common hematologic abnormality among critically ill patients and frequently occurs in the setting of sepsis, typically defined as a platelet count below 150 × 10⁹/L [7]. Platelets are essential to normal hemostasis, contributing to vascular injury recognition, platelet adhesion and aggregation, and the formation of a stable hemostatic clot [8]. Consequently, significant reductions in circulating platelets can disrupt normal hemostasis and increase the risk of bleeding [7,8]. In critically ill patients, thrombocytopenia is clinically significant not only because of the associated bleeding risk but also because it can serve as an important marker of disease severity [7,9]. Studies have reported that thrombocytopenia develops in approximately 14-44% of patients admitted to the intensive care unit, although the reported incidence varies according to the population studied and the criteria used [7]. Among patients with sepsis, thrombocytopenia is particularly common and has been associated with greater disease severity and worse clinical outcomes [4,9].

The development of thrombocytopenia during sepsis is often multifactorial, making the underlying etiology difficult to determine [9,9]. Sepsis itself can contribute to platelet consumption and destruction through systemic inflammation, endothelial activation, and coagulation abnormalities; however, other potentially life-threatening conditions may present similarly [4,9]. Important considerations include DIC, ITP, and TMAs, including TTP and HUS [4]. DIC is particularly common in sepsis, but overlapping clinical and laboratory findings can make it difficult to distinguish from TMA and other causes of thrombocytopenia [4]. Current literature therefore emphasizes evaluating these disorders concurrently rather than attributing thrombocytopenia to sepsis or DIC alone [4]. Accurate differentiation is essential because the underlying mechanisms, treatment approaches, and potential complications differ substantially among these conditions [4].

Pathology/pathophysiology 

Sepsis-associated thrombocytopenia develops through multiple overlapping mechanisms, including systemic inflammation, endothelial activation, platelet activation, and increased peripheral platelet consumption and destruction [4,9]. In DIC, widespread activation of the coagulation cascade leads to excessive thrombin generation and fibrin deposition, resulting in consumption of platelets and coagulation factors and contributing to both bleeding and microvascular thrombosis [10]. In contrast, ITP is primarily caused by immune-mediated destruction of circulating platelets, with compensatory increased platelet production by the bone marrow; therefore, an elevated immature platelet fraction may support a peripheral destructive process [11]. TMAs, including TTP and HUS, are characterized by platelet-rich microvascular thrombi and microangiopathic hemolytic anemia, which is typically associated with schistocytes, elevated lactate dehydrogenase, and decreased haptoglobin [4,12]. Given the clinical overlap among these disorders in patients with sepsis, distinguishing between them requires careful interpretation of the peripheral blood smear, hemolysis markers, coagulation studies, and platelet production indices [4,10,11]. In this case, the absence of significant schistocytosis and convincing evidence of hemolysis argued against a TMA, while the overall clinical picture was more consistent with sepsis-associated thrombocytopenia. 

Literature review/comparative analysis 

Clinical Presentation

Actinotignum schaalii is increasingly recognized as an invasive uropathogen, particularly in older adults with underlying structural or functional abnormalities of the genitourinary tract [2,13-15]. Reported predisposing factors include benign prostatic disease, urinary obstruction, chronic urinary retention, urinary instrumentation, and other conditions that impair normal urinary drainage [2,13-15]. Bacteremia most frequently arises from a urinary source, and pyelonephritis has been identified as a common clinical presentation among patients with invasive A. schaalii infection [2,13]. The demographic and urologic characteristics of our patient therefore resemble those described in the existing literature: an older man with marked bladder outlet pathology, chronic urinary instrumentation, severe unilateral hydroureteronephrosis, and bloodstream infection.

The distinguishing feature of the present case was the development of profound thrombocytopenia accompanied by acute kidney injury and systemic inflammation. Thrombocytopenia occurs frequently during sepsis and may result from multiple simultaneous mechanisms, including platelet activation, peripheral consumption, immune-mediated destruction, endothelial injury, and activation of coagulation pathways [4]. Severe thrombocytopenia combined with renal dysfunction may closely resemble DIC or TMA, including TTP and HUS [4]. Published reports of A. schaalii bacteremia have focused predominantly on infectious and urologic manifestations, whereas profound thrombocytopenia producing this degree of diagnostic uncertainty has been sparsely characterized [2,3,13-15].

Diagnostic Workup

Diagnosis of A. schaalii infection can itself be challenging because the organism is fastidious, slow-growing, and may not be recovered under conventional urine culture conditions [2,13-15]. Modern identification techniques, including matrix-assisted laser desorption/ionization time-of-flight mass spectrometry, have substantially improved recognition of this organism in clinical specimens [13,14]. Importantly, negative urine cultures do not exclude a urinary source of A. schaalii bacteremia, and previous reports have documented bloodstream infection despite failure to recover the organism from urine [3]. This pattern closely paralleled our patient, whose blood cultures yielded A. schaalii despite a negative urine culture.

The more consequential diagnostic dilemma involved determining the mechanism of thrombocytopenia. Contemporary approaches recommend evaluating DIC and TMA concurrently in septic patients with severe thrombocytopenia rather than automatically attributing the platelet reduction to sepsis-associated DIC [4]. TMA is typically characterized by thrombocytopenia accompanied by microangiopathic hemolytic anemia, with findings such as schistocytes, increased lactate dehydrogenase, reduced haptoglobin, and evidence of organ injury [4]. DIC, in contrast, is associated with systemic coagulation activation, platelet and coagulation-factor consumption, and characteristic abnormalities in coagulation parameters and fibrin-related markers [4]. In our patient, the absence of significant schistocytosis and convincing biochemical evidence of hemolysis argued against TMA, whereas preserved or elevated fibrinogen and relatively modest coagulation abnormalities made overt consumptive DIC less likely. The markedly elevated immature platelet fraction suggested preserved marrow platelet production and favored peripheral consumption or destruction, although this finding alone cannot reliably differentiate infection-associated thrombocytopenia from an immune-mediated process [4].

Management

Management of invasive A. schaalii infection requires appropriate antimicrobial therapy together with correction or management of the underlying urinary tract pathology when clinically indicated [2,13-15]. The organism is generally susceptible to beta-lactam antibiotics, whereas reduced susceptibility or resistance to commonly prescribed urinary agents, including ciprofloxacin and trimethoprim-sulfamethoxazole, has been described [2-4]. Accurate organism identification is therefore important not only diagnostically but also for selection of effective antimicrobial therapy [13-15]. In the present case, broad-spectrum intravenous antibiotics were appropriate during the initial septic presentation, with subsequent microbiologic identification permitting refinement of the infectious diagnosis.

The hematologic management required greater caution because therapies for DIC, TTP/HUS, ITP, and nonspecific sepsis-associated thrombocytopenia differ substantially [4]. Current literature emphasizes establishing evidence for microangiopathic hemolysis and coagulation consumption before committing patients to disease-specific interventions such as therapeutic plasma exchange [4]. The absence of convincing evidence for TMA or overt DIC in our patient allowed management to remain directed toward the underlying infection and supportive care while hematologic parameters were followed serially.

Outcome and Follow-Up

Bloodstream infection with A. schaalii occurs predominantly in older patients with substantial comorbidity and can be associated with prolonged hospitalization and clinically significant mortality [14]. Severe systemic infection and septic shock have also been reported, confirming that this organism should not be regarded merely as a low-virulence urinary colonizer [3,14]. Our patient demonstrated a favorable course, with clinical improvement, clearance of bacteremia, normalization of renal function, and substantial recovery of the platelet count during hospitalization. The parallel improvement of thrombocytopenia with control of the infectious process further supported an infection-associated mechanism rather than an untreated progressive TMA. Nevertheless, longer-term hematologic follow-up would be valuable because improvement during a single hospitalization cannot definitively exclude an underlying immune-mediated thrombocytopenic disorder. Table 2 summarizes the comparative analysis section. 

**Table 2:** Comparison of the Present Case With Existing Literature on Actinotignum schaalii Bacteremia and Sepsis-Associated Thrombocytopenia Comparison of the clinical presentation, diagnostic evaluation, management, and outcome of the present case with findings reported in contemporary literature on Actinotignum schaalii infection and thrombocytopenia in sepsis. The patient's demographic and urologic risk factors were characteristic of previously reported invasive A. schaalii infections; however, the profound thrombocytopenia with concomitant acute kidney injury produced an unusual diagnostic overlap among sepsis-associated thrombocytopenia, DIC, TMA, TTP/HUS, and ITP. Abbreviations: BPH, benign prostatic hyperplasia; DIC, disseminated intravascular coagulation; HUS, hemolytic uremic syndrome; ITP, immune thrombocytopenia; LDH, lactate dehydrogenase; TMA, thrombotic microangiopathy; TTP, thrombotic thrombocytopenic purpura.

Clinical Domain	Findings Reported in Existing Literature	Present Case	Clinical Significance
Typical patient population	A. schaalii infection occurs predominantly in older adults, particularly men with underlying urologic abnormalities [2,13-15].	73-year-old man with significant chronic genitourinary disease.	Consistent with the demographic profile of previously reported invasive A. schaalii infections.
Predisposing urinary abnormalities	BPH, urinary obstruction, chronic retention, urinary instrumentation, and structural urinary tract abnormalities are commonly reported [2,13-15].	Chronic bladder outlet obstruction, suprapubic catheter, prostatomegaly, and severe left hydroureteronephrosis.	Strongly supports the urinary tract as the likely source of bacteremia.
Infectious presentation	Complicated urinary tract infection, pyelonephritis, bacteremia, and occasionally septic shock have been described [2,3,13-15].	Systemic infection with A. schaalii bacteremia and evidence of complicated urinary tract disease.	Similar infectious phenotype to previously reported invasive cases.
Urine culture	Conventional urine cultures may fail to identify A. schaalii because of its fastidious growth requirements [2,3,13-15].	Urine culture demonstrated no growth despite positive blood cultures.	Illustrates the potential diagnostic limitation of routine urine cultures.
Blood cultures	Blood cultures can identify A. schaalii in patients with invasive urinary infection [2,3,13-15].	Two blood culture sets yielded gram-positive rods subsequently identified as A. schaalii.	Established the microbiologic diagnosis despite negative urine culture.
Thrombocytopenia	Thrombocytopenia is common in sepsis and may reflect peripheral consumption, immune-mediated destruction, endothelial activation, or DIC [4].	Profound thrombocytopenia with platelet count approximately 16 ×10³/µL.	Severity prompted evaluation beyond uncomplicated sepsis-associated thrombocytopenia.
DIC consideration	Sepsis-associated DIC may produce thrombocytopenia, coagulation abnormalities, increased fibrin-related markers, and consumption of coagulation factors [4].	Elevated D-dimer with relatively modest coagulation abnormalities and preserved/elevated fibrinogen.	Findings did not establish overt consumptive DIC.
TMA/TTP-HUS consideration	TMA typically presents with thrombocytopenia, microangiopathic hemolytic anemia, schistocytes, increased LDH, decreased haptoglobin, and organ injury [4].	Acute kidney injury and severe thrombocytopenia were present, but significant schistocytosis and convincing biochemical hemolysis were absent.	Reduced the likelihood of TTP/HUS-spectrum thrombotic microangiopathy.
Platelet production	Increased immature platelet indices may indicate preserved marrow production with peripheral platelet consumption or destruction [4].	Immature platelet fraction markedly elevated at 39%.	Supported a peripheral mechanism rather than impaired platelet production, although it was not etiologically specific.
Renal dysfunction	Acute kidney injury may accompany both severe sepsis and TMA [4].	Creatinine was 2.40 mg/dL on presentation; imaging also revealed severe chronic left obstructive nephropathy with 14.5% differential function.	Demonstrated that renal impairment had both acute and chronic contributors and could not independently establish TMA.
Antimicrobial management	Beta-lactam agents generally demonstrate activity against A. schaalii, whereas resistance or reduced susceptibility to some commonly used urinary antimicrobials has been reported [13-15].	Broad-spectrum intravenous antimicrobial therapy was initiated during the septic presentation and adjusted according to microbiologic findings.	Consistent with management of severe invasive infection while awaiting definitive identification.
Hematologic management	Disease-specific treatment for DIC or TMA should be guided by the complete clinical and laboratory pattern rather than thrombocytopenia alone [4].	Serial smear, hemolysis, coagulation, platelet, and renal assessments were used to avoid premature TMA- or DIC-directed therapy.	Highlights the importance of parallel rather than sequential evaluation of competing etiologies.
Clinical outcome	Invasive A. schaalii infection may cause substantial morbidity and mortality in older, comorbid patients [3,14].	Bacteremia resolved, creatinine improved to approximately 0.81 mg/dL, and platelet count recovered to 341 ×10³/µL.	Favorable response supported infection-associated thrombocytopenia rather than an untreated progressive TMA.
Distinguishing contribution of this case	Published A. schaalii reports primarily emphasize urinary infection, bacteremia, and urologic risk factors [2,3,13-15].	Profound thrombocytopenia simultaneously raised concern for sepsis-associated thrombocytopenia, DIC, TMA/TTP-HUS, and ITP.	Adds a hematologic diagnostic perspective that is poorly characterized in the existing A. schaalii literature.

What We Learned From This Case

This case highlights the diagnostic complexity of severe thrombocytopenia occurring in the setting of Actinotignum schaalii bacteremia. Although sepsis-associated thrombocytopenia is common, profound thrombocytopenia accompanied by acute kidney injury and systemic inflammation should prompt a broad differential diagnosis that includes DIC, TMA (TTP/HUS), immune-mediated thrombocytopenia, and other consumptive processes. Premature attribution of thrombocytopenia to sepsis alone may delay appropriate investigation and management.

An important lesson from this case is the value of integrating clinical presentation with serial laboratory testing rather than relying on a single abnormal finding. Preserved fibrinogen levels, the absence of significant hemolysis, stable coagulation parameters, and progressive platelet recovery following treatment of the underlying infection supported a diagnosis of sepsis-associated thrombocytopenia rather than DIC or TMA. Likewise, advanced imaging demonstrated longstanding unilateral obstructive nephropathy with severely reduced differential renal function, indicating chronic irreversible renal disease rather than acute bilateral renal injury as the primary explanation for the patient's kidney dysfunction.

Finally, this case expands the limited literature describing A. schaalii bacteremia and underscores that this emerging uropathogen can present with severe systemic illness and hematologic abnormalities that closely mimic life-threatening thrombotic and consumptive disorders. Recognition of these diagnostic pitfalls and a multidisciplinary approach are essential to avoid unnecessary interventions while ensuring timely, targeted treatment.

Limitations

This report has several limitations. First, as a single-patient case report, the observations cannot establish a causal relationship between Actinotignum schaalii bacteremia and the severe thrombocytopenia, and the association should be interpreted with caution. Second, although the diagnostic evaluation was comprehensive, the diagnosis of sepsis-associated thrombocytopenia was made by exclusion after careful assessment for DIC, TMA, and ITP rather than confirmation with a definitive diagnostic test. Finally, long-term hematologic follow-up was unavailable; therefore, recurrence of thrombocytopenia or the subsequent development of an alternative hematologic disorder could not be assessed.

## Conclusions

This case highlights the diagnostic complexity of severe thrombocytopenia in the setting of Actinotignum schaalii bacteremia. The simultaneous presence of profound thrombocytopenia, acute kidney injury, and systemic inflammation initially suggested life-threatening hematologic disorders, including DIC and TMA. However, a systematic evaluation integrating microbiologic, hematologic, and radiographic findings demonstrated sepsis-associated thrombocytopenia superimposed on chronic obstructive nephropathy rather than a primary thrombotic or consumptive process. Recognition of the patient's underlying chronic genitourinary abnormalities and careful exclusion of competing diagnoses allowed appropriate antimicrobial therapy and supportive management while avoiding unnecessary interventions. As A. schaalii is increasingly recognized as an important uropathogen in older adults with urinary tract abnormalities, clinicians should maintain a broad differential diagnosis and integrate clinical, laboratory, and imaging findings to accurately identify the cause of thrombocytopenia and guide timely management.
